# Knowledge, attitudes, and practices of Sudanese medical professionals regarding the 2023 Sudan malaria treatment protocol: a cross-sectional study

**DOI:** 10.1186/s12936-026-06064-y

**Published:** 2026-07-22

**Authors:** Yousif Omer Elgaili Yousif, Rayan Mohamed Issa Kheerallah, Maab Khalifa Mohamed Ahmed, Khadiga Mansoor Ali Bakheit, Azza Omer Osman Abdelrahman, Hind Elmukashfi ShamsEldin Elobied, Sittia Basil Hassan Ali, Srraa Abdelrasoul Elnour Ismail, Sahl Muawia Elbsri Elshaikh, Tarik Abdulrahim Ahmed Yousif, Adam Elnour Mohamed Ibrahim, Ahmed Eltayeb Ali Hassan, Mahmoud Alballa Almahdi Alhag, Faisal Rhma Ismail Adam, Omer Elgaili Yousif Elhag, Mohammed Mahmmoud Fadelallah Eljack

**Affiliations:** 1 Alzaiem Alazhari University, Khartoum, Sudan; 2 University of Khartoum, Khartoum, Sudan; 3 Ahfad University for Women, Khartoum, Sudan; 4 International University of Africa, Khartoum, Sudan; 5 University of Science and Technology, Khartoum, Sudan; 6 National University of Sudan, Khartoum, Sudan; 7 West Kordofan University, West Kordofan, Sudan; 8 University of Gezira, Gezira, Sudan; 9 University of Al Fashir, Al Fashir, Sudan; 10 Omdurman Islamic University, Khartoum, Sudan; 11 Department of Internal Medicine, Faculty of Medicine, Al Neelain University, Khartoum, Sudan; 12 Faculty of Medicine and Health Sciences, University of Bakht Alruda, Ad Duwaym, Sudan

## Abstract

**Introduction:**

Malaria remains a major public health challenge in Sudan, primarily driven by Plasmodium falciparum. Timely and effective case management is critical, necessitating high adherence to the newly issued Sudan Malaria Case Management Protocol 2023. This study aimed to assess the knowledge, attitudes, and current practices of Sudanese medical professionals regarding this 2023 protocol.

**Methodology:**

This was a cross-sectional analytical study conducted in 2024–2025. A convenience sample of 371 currently practicing Sudanese medical professionals was recruited and surveyed. Data were analyzed to identify levels of awareness and adherence, as well as the major barriers to protocol implementation.

**Results:**

High awareness was reported, with 67.9% of participants knowing about the 2023 protocol. Knowledge of first-line treatments was strong, with 93.5% correctly identifying Artemether-Lumefantrine for uncomplicated malaria and 83.0% correctly identifying Intravenous Artesunate for severe malaria. However, a significant gap in formal training was observed: 69.5% of participants reported no formal training on the new protocol. Furthermore, only 41.5% reported "always" adhering to the protocol in their practice. The major reported barriers included lack of formal training (55.8%) and lack of resources (53.4%).

**Conclusion:**

Although the 2023 protocol is well-regarded as an improvement, and knowledge of key recommendations is high, its potential is limited by significant implementation challenges. Translating awareness into effective clinical practice requires immediate, comprehensive interventions, including structured training and addressing resource shortages.

## Introduction

In Sudan, malaria is a serious public health issue. Malaria accounted for the majority of hospital admissions, inpatient fatalities, and outpatient visits in Sudan in 2021. More than 87.6% of cases are caused by the most prevalent infectious species, Plasmodium falciparum (P. falciparum). 8.1% of cases were caused by Plasmodium vivax (P. vivax), whereas 4.3% were caused by mixed infections (P. falciparum and P. vivax). However, recent statistical reports suggest that the present prevalence of P. vivax is higher than previously thought. Early malaria identification and treatment are critical, as highlighted by the Sudan Malaria Case Management Protocol 2023 [1].

High rates of morbidity and mortality are caused by malaria, particularly in children under five. Even though the global malaria fatality rate has decreased by more than 25% and by 33% in the WHO African Region, malaria is still transmitted in 99 countries. Children under the age of five constituted the majority of those afflicted by malaria in 2020, with a projected 241 million cases and 627,000 malaria fatalities occurring globally. Providing appropriate care for individuals who may have malaria is a crucial strategy for managing the disease [2].

Worldwide progress in controlling malaria has improved significantly, but in recent years, it has plateaued. Since 2010, the 'test and treat policy,' which involves treating only test-positive cases with artemisinin-based combination therapy (ACT) and managing cases of suspected malaria based on parasitological confirmation of all suspected patients, has been a mainstay of malaria control in Africa. While the availability of "test and treat" commodities is a necessary condition for the policy's execution, health professionals' adherence to advised protocols establishes the parameters for efficient surveillance and assesses the case-management program's cost-effectiveness. There have been recent reports of advances in malaria "test and treat" compliance in African health institutions [3].

The results of the treatment efficacy study led to the most recent revision of Sudan's malaria case management policy in 2017. The effectiveness of artesunate + sulphadoxine-pyremethamine (AS + SP) was shown to be declining, especially in Gedarif State, where treatment failure rates were higher than 10%. Additionally, the study showed that dihydroartemisinin-piperaquine (DHAP) and artemether-lumefantrine (AL) were highly effective (> 95%). Additionally, the 2016 Sudan Malaria Indicators Survey found that injectable artemether was being used irrationally to treat simple malaria. These results led to the recommendation of AL and DHAP as first and second-line treatments for uncomplicated malaria, respectively. The Federal Ministry of Health continues to support the previously outlined proposals. However, an update for the treatment of severe malaria in Sudan was suggested by the Malaria Program Review 2018. To update the Sudan Malaria Protocol and its associated training materials, the National Malaria Control Program (NMCP) summoned a meeting of the Technical Advisory Committee (TAC). The Sudan Malaria Case Management Protocol 2023 was created using the TAC recommendations and the WHO Guidelines for Malaria, which were released on November 25, 2022 [1].

Enhancing the standard of clinical case management for malaria in healthcare institutions is essential to enhancing patient outcomes. Seeking for a professional health care system involving the applying of protocols in Sudan was a big problem facing Sudanese health professionals; since that assessing the knowledge, attitude and practice to the newly developed protocols to assess the application of such important guidelines was very important to improve the health system and the patient’s satisfaction toward their treatments and health and improve the patient doctor relationships.

Data on the application of such protocols in Sudan are scarce. This cross-sectional survey was conducted with the aim of assessing medical professionals' knowledge, attitudes, and practices regarding the 2023 Sudan malaria treatment protocol.

## Methodology

### Study design

A cross-sectional descriptive study.

### Study duration

It took one month after proposal approval.

### Study population

This study focuses on Sudanese House officers, medical officers, residents, specialists. Participants were not required to have received formal training on the protocol prior to participation. The survey assessed both formally trained and non-trained healthcare professionals.

### Inclusion criteria

Doctors currently practicing Medicine inside Sudan.

Doctors having a practice license from the Sudan Medical Council.

### Exclusion criteria

Other hospital staff.

Medical students or other health sector students.

### Independent variable

Sudan Malaria Treatment Protocol 2023: This is the primary factor influencing the perceptions studied. It's considered independent because it exists regardless of the medical professionals' views.

### Dependent variable

Knowledge.

Attitudes.

Practices.

### Sample size

The sample size is calculated with 5% deviation and 95% confidence interval and anticipated frequency (response distribution) of 50% using OpenEpi sample size calculator.

$$Sample\,size\,n\, = \,\left[ {DEFF^{ * } } \right.Np\left. {\left( {1 - p} \right)} \right]\,/\,\left[ {\left( {{{d^{2} } \mathord{\left/ {\vphantom {{d^{2} } {Z^{2} }}} \right. \kern-0pt} {Z^{2} }}} \right.} \right._{{1 - {\alpha \mathord{\left/ {\vphantom {\alpha 2}} \right. \kern-0pt} 2}}}^{ * } \left( {N - 1} \right)\, + \,\left. {\left( {1 - p} \right)} \right]$$

Population size for finite population correction factor or fpc (N): 10000.

Hypothesized % frequency of outcome factor in the population (p): 50% ± 5.

Confidence limits as % of 100(absolute ± %)(d): 5%

Design effect (for cluster surveys-DEFF):1

The sample size is 370.

### Sampling technique

A non-probability convenience sampling was done for all house officers, medical officers, registrars and consultants within Sudanese teaching and referral hospitals. The form was distributed to 20 participants in a pilot study to assess how each participant perceived the questions.

### Statistical analysis

Microsoft Excel was used to enter the data that had been gathered. IBM SPSS Statistics (Version 27) was used to perform statistical analysis.

### Consent and ethical approval

The study was approved by the Federal Ministry of Health and complied with ethical criteria. Throughout the research process, every precaution was taken to guarantee patient confidentiality and data protection. All patient data was anonymized, and personal identifiers were eliminated from the dataset to ensure confidentiality. This method protected patient privacy and adhered to ethical standards. All gathered data was kept in a password-protected electronic database with restricted access for data protection. To protect the data's integrity and stop illegal access or misuse, only authorized personnel could handle it. The STROBE standards for observational studies have been followed in the reporting of this work.

## Results

In this study, 371 medical professionals participated. The demographic profile of the participants is shown in Table 1. The majority of participants (68.5%, n = 254) were aged 25–30. Majority of participants were female (53.4%, n = 198).

**Table 1:** Demographic Data

Variable	Category	Frequency	Percent
Age	18–24	66	17.8
	25–30	254	68.5
	30–40	45	12.1
	40–50	5	1.3
	more than 50	1	0.3
Gender	Female	198	53.4
	Male	172	46.4
	Prefer not to say	1	0.3
Professional role	General Practitioner	147	39.6
	House officers	173	46.6
	Registerer	43	11.6
	Specialist Doctor	8	2.2
Years of experience	11–20 years	15	4.0
	5–10 years	49	13.2
	Less than 5 years	307	82.7
Type of health care facility	Community health center	13	3.5
	Government hospital	314	84.6
	Mobile clinic	3	0.8
	No practice	1	0.3
	Nothing	1	0.3
	Obs and gyn	1	0.3
	Pharmacist	1	0.3
	Private clinic	26	7.0
	Rural health post	11	3.0
Location of The practice	Rural	80	21.6
	Suburban	63	17.0
	Urban	228	61.5
Working in malaria-endemic area	Maybe	29	7.8
	No	58	15.6
	Yes	284	76.5

House officers made up the largest group in terms of professional roles (46.6%, n = 173), followed by general practitioners (39.6%, n = 147), registrars (11.6%, n = 43), and specialist physicians (2.2%, n = 8). Of the participants, most of them 82.7% (n = 307) had less than 5 years of professional experience.

The majority of participants (84.6%, n = 314) were employed by government hospitals according to the geographic distribution of practice locations. Interestingly, 76.5% of participants (n = 284) said they worked in areas where malaria was endemic.

The study evaluated the participants' knowledge and comfort level with the Sudan Malaria Treatment Protocol for 2023. According to Table 2, 67.9% (n = 252) of participants said they were aware of the 2023 protocol, compared to 11.9% (n = 44) who were not and 20.2% (n = 75) who were unsure. In comparison to the previous protocol, 59.0% (n = 219) of respondents said they were somewhat familiar with the 2023 protocol, 29.4% (n = 109) said they were very familiar, and 11.6% (n = 43) said they were not familiar.

**Table 2:** Awareness of Sudan malaria treatment protocol 2023

Variable	Category	Frequency	Percent
Awareness of the 2023 protocol	Maybe	75	20.2
	No	44	11.9
	Yes	252	67.9
Familiarity of the 2023 protocol compared to the previous protocol	Not familiar	43	11.6
	Somewhat familiar	219	59.0
	Very familiar	109	29.4

The vast majority of participants thought the protocol sufficiently addressed the needs of children under five years old (75.7%, n = 281) and pregnant women (77.9%, n = 289). Fewer participants, however, (61.7%, n = 229) thought the protocol sufficiently addressed the needs of patients with co-morbidities. Interestingly, only 27.0% (n = 100) of participants thought the protocol met the needs of HIV/AIDS patients. 66.3% (n = 246) of participants said that the protocol adequately addresses all patient groups. As shown in Table 3

**Table 3:** The addressing of the 2023 protocol to the needs of special population

Variable	Category	Frequency	Percent
Pregnant women	Maybe	41	11.1
	No	41	11.1
	Yes	289	77.9
Children under 5 years	Maybe	43	11.6
	No	47	12.7
	Yes	281	75.7
HIV/AIDS	Maybe	71	19.1
	No	200	53.9
	Yes	100	27.0
Patients with co-morbidities	Maybe	67	18.1
	No	75	20.2
	Yes	229	61.7
All patient groups are addressed adequately	Maybe	92	24.8
	No	33	8.9
	Yes	246	66.3

49.6% (n = 184) of participants agreed that 2023 protocol is an improvement over the previous one. 71.5% of participants overall expressed confidence in the efficacy of the protocol, with 27.0% (n = 100) expressing very high confidence. 72.8% of participants thought the new protocol was practical when it came to implementing it in the current healthcare setting. Of those, 41.0% (n = 152) thought it was somewhat practical, and 31.8% (n = 118) thought it was very practical.

Artemether-Lumefantrine (Coartem) was correctly identified as the suggested first-line treatment by the great majority of participants (93.5%, n = 347). There are some knowledge gaps among healthcare professionals, as evidenced by the small percentage who named Artesunate-Amodiaquine (ASAQ) (3.2%, n = 12), Chloroquine (1.3%, n = 5), or Quinine (1.3%, n = 5) as the first-line treatment. As shown in Table 4.

**Table 4:** Attitudes toward the 2023 Sudan malaria treatment protocol and knowledge of the recommended first-line treatment

	Frequency	Percent
Agreeing that the 2023 Sudan Malaria Treatment Protocol is an improvement over the previous protocol:		
Strongly agree	66	17.8
Agree	184	49.6
Neutral	108	29.1
Disagree	12	3.2
Strongly disagree	1	0.3
Confidence of the effectiveness of the new protocol in reducing malaria-related mortality and morbidity:		
Very confident	100	27
Somewhat confident	165	44.5
Neutral	93	25.1
Not confident	12	3.2
Very unconfident	1	0.3
Very confident	100	27
Practicality of the implementation of the new protocol in the current healthcare setting:		
Very practical	118	31.8
Somewhat practical	152	41.0
Neutral	85	22.9
Not practical	13	3.5
Very impractical	3	0.8
Recommended antimalarial drugs as the first-line treatment for uncomplicated malaria according to the 2023 protocol:		
Artemether-Lumefantrine (Coartem)	347	93.5
Artesunate-Amodiaquine (ASAQ)	12	3.2
Chloroquine	5	1.3
Coartem	1	0.3
Quinine	5	1.3
Don’t know	1	0.3

Table 5 shows that a significant majority of participants (69.5%, n = 258) reported not having received formal training on the 2023 Sudan Malaria Treatment Protocol, despite the protocol's generally positive reception. As suggested by the 2023 protocol, Table 5 also shows difficulties in tracking and disclosing side effects after treatment. Nearly half (47.2%, n = 175) said that regular monitoring and reporting of negative effects was not being done. The availability of key resources for managing malaria is shown in Table 5. 95.1% (n = 353) of participants confirmed that first-line antimalarial medications were widely accessible. Similarly, 85.7% (n = 318) of participants reported having access to intravenous medications for severe malaria. 81.4% (n = 302) of participants reported having access to trained personnel and microscopy, while 73.0% (n = 271) reported having rapid diagnostic tests (RDTs).

**Table 5:** Training, resource availability, monitoring practices, and adherence to the 2023 Sudan Malaria Treatment Protocol

	Response	Frequency	Percent
Receiving a formal training on the 2023 Sudan Malaria Treatment Protocol	Maybe	23	6.2
	No	258	69.5
	Yes	90	24.3
Consistent monitoring and reporting of the adverse effects following treatment as recommended in the 2023 protocol	Maybe	77	20.8
	No	175	47.2
	Yes	119	32.1
Availability of resources:			
First-line antimalaria drugs	No	18	4.9
	Yes	353	95.1
IV medications for severe malaria	No	53	14.3
	Yes	318	85.7
Rapid diagnostic test (RDT)	No	100	27.0
	Yes	271	73.0
Microscopy and trained personnel	No	69	18.6
	Yes	302	81.4
Insecticide-treated nets	No	153	41.2
	Yes	218	58.8
Adherence to the 2023 protocol:			
When treating patients	Always	154	41.5
	Never	15	4.0
	Often	114	30.7
	Rarely	16	4.3
	Sometimes	72	19.4
When collaborating with other healthcare workers	Always	85	22.9
	Never	30	8.1
	Often	105	28.3
	Rarely	43	11.6
	Sometimes	108	29.1

However, only 58.8% (n = 218) of participants confirmed the availability of insecticide-treated nets, which are essential for preventing malaria, while a significant 41.2% (n = 153) reported that they were not available. Data on healthcare professionals' compliance with the 2023 protocol is shown in Table 5. 41.5% (n = 154) said they always did so when treating patients. While when working with other healthcare professionals, there was reportedly less adherence to the protocol. In collaborative settings, A significant 29.1% (n = 108) said they followed the protocol occasionally.

Healthcare professionals' awareness of the 2023 protocol's suggested malaria diagnostic techniques is shown in Table 6. The most common diagnostic technique was microscopy (75.7% of respondents, n = 281), which was followed by the Rapid Diagnostic Test (RDT) (49.9%, n = 185). Interestingly, despite the protocol's emphasis on parasitological confirmation, a significant percentage of participants (39.4%, n = 146) recognized clinical diagnosis based on symptoms as a recommended diagnostic method. Lesser percentages of participants recognized the immunochromatographic test (ICT) for malaria (9.2%, n = 34) and polymerase chain reaction (PCR) (10.5%, n = 39) as suggested diagnostic techniques. Just 0.3% of participants (n = 1) were able to identify blood film for malaria, and 0.5% (n = 2) said they were unaware of the suggested diagnostic techniques.

**Table 6:** Knowledge of recommended diagnostic methods, treatment of severe malaria, preventive measures, and perceived barriers and challenges to implementation of the 2023 Sudan Malaria Treatment Protocol

	Responses	N	Percent
Recommended Measures for Malaria According to the 2023 Protocol:			
Recommended Diagnostic Method	Blood Film for Malaria	1	0.1
	Clinical diagnosis based on symptoms	146	21.2
	No idea	2	0.3
	Microscopy	281	40.8
	ICT for malaria	34	4.9
	Polymerase Chain Reaction (PCR)	39	5.7
	Rapid Diagnostic Test (RDT)	185	26.9
Antimalarials For Complicated Malaria	Intramuscular Artemether	25	4.4
	Intravenous Artesunate	308	54.2
	Intravenous Quinine	161	28.3
	Oral Artemether-Lumefantrine after parenteral therapy	73	12.9
	Don't know	1	0.2
Preventive Measures In 2023 Protocol	Indoor residual spraying (IRS)	218	29.7
	Intermittent preventive treatment (IPT) in pregnancy	106	14.5
	Mass drug administration (MDA)	69	9.4
	Insecticide-treated bed nets (ITNs)	335	45.7
	Chemotherapy for travel	1	0.1
	Malaria vaccines	2	0.3
	Don't know	2	0.3
Barriers and challenges against adoption and adherence of the 2023 protocol:			
Barriers Against 2023 Protocol Adoption	Complexity of the protocol	58	8.9
	Patient non-compliance	181	27.9
	Don’t know	1	0.2
	Lack of resources (e.g., medications, diagnostic tools)	198	30.5
	Recurrence	1	0.2
	Lack of training	207	31.9
	Injection preference	1	0.2
	Wide resistance	1	0.2
	Monitor and supervision	1	0.2
Challenges Against Adherence to Protocol	Don’t know	1	0.2
	Lack of patient follow-up	241	37.0
	Limited access to diagnostic tools	131	20.1
	Time constraints	89	13.7
	Limited access to recommended medications	183	28.1
	High cost of the medications	1	0.2
	Injections are preferred	1	0.2
	Resistance to medication	2	0.3
	Nurse negligence	1	0.2
	None	1	0.2

Because participants were able to choose from a variety of diagnostic techniques, the overall percentage surpassed 100% (185.4%), suggesting that the majority of medical professionals were aware of multiple suggested techniques.

Also, Table 6 shows how well-informed medical professionals are about the 2023 protocol's suggested course of treatment for severe malaria. A significant majority of participants (83.0%, n = 308) correctly identified intravenous artesunate as a recommended treatment for severe malaria. Nonetheless, a sizeable percentage (43.4%, n = 161) also recognized intravenous quinine, indicating some misunderstanding or awareness of substitute therapies. 19.7% (n = 73) of participants identified oral Artemether-Lumefantrine following parenteral therapy, while 6.7% (n = 25) identified intramuscular Artemether. Just one person (0.3%) said they were unaware of the best course of action for treating severe malaria.

The fact that participants could choose from a variety of treatment options means that, like the diagnostic methods, the overall percentage is higher than 100% (153.1%), suggesting that many medical professionals were aware of multiple recommended treatments for severe malaria.

Healthcare professionals' awareness of the 2023 protocol's suggested malaria preventive measures is shown in Table 6. The majority of participants (90.3%, n = 335) recognized insecticide-treated bed nets (ITNs) as a suggested preventive measure. There was also broad recognition of indoor residual spraying (IRS) (58.8%, n = 218). Of the participants, 28.6% (n = 106) had identified intermittent preventive treatment (IPT) during pregnancy, and 18.6% (n = 69) had identified mass drug administration (MDA). Just 0.5% (n = 2) of participants said they were unaware of the recommended preventive measures, and only a small percentage of participants recognized chemotherapy for travel (0.3%, n = 1) and malaria vaccines (0.5%, n = 2) as suggested preventive measures.

Because participants could choose more than one preventive measure, the overall percentage significantly surpasses 100% (197.6%), suggesting that the majority of medical professionals were aware of multiple malaria preventive measures.

Healthcare professionals' opinions of the obstacles impeding the adoption of the 2023 Sudan Malaria Treatment Protocol are shown in Table 6. A major obstacle cited by 55.8% of respondents (n = 207) was a lack of training, which was followed by a lack of resources, including drugs and diagnostic equipment (53.4%, n = 198). Nearly half of the participants (48.8%, n = 181) also cited patient non-compliance as a significant obstacle. A lower percentage of participants (15.6%, n = 58) said that the protocol's complexity was a barrier. Individual participants pointed to a number of additional obstacles, such as recurrence, injection preference, widespread resistance, and a lack of oversight and monitoring (0.3%, n = 1 each).

Because participants could choose more than one barrier, the overall percentage surpassed 100% (174.9%), suggesting that the majority of medical professionals identified multiple factors preventing the protocol's adoption.

The difficulties faced by medical personnel in trying to follow the 2023 protocol are shown in Table 6. The biggest obstacle was found to be a lack of patient follow-up (65.0% of respondents, n = 241), which was followed by restricted access to prescribed drugs (49.3%, n = 183) and diagnostic instruments (35.3%, n = 131). About a quarter of participants (24.0%, n = 89) cited time constraints as a challenge. Few participants mentioned a number of other difficulties, such as medication resistance (0.5%, n = 2), the high expense of medications, injection preference, nurse carelessness, and the absence of difficulties (0.3%, n = 1 each).

The fact that participants could choose more than one challenge means that, like barriers, the overall percentage is higher than 100% (175.5%), suggesting that most healthcare professionals encounter multiple challenges when trying to follow the 2023 protocol.

The recommendations of medical experts for enhancing the 2023 Sudan Malaria Treatment Protocol are shown in Table 7. 62.5% of participants (n = 232) recommended improved patient education materials and updated medication guidelines. 58.8% (n = 218) recommended better diagnostic testing practices, 52.3% (n = 194) recommended more explicit treatment protocols for complex cases, and 50.9% (n = 189) recommended improved medication supply chain management. Individual participants (0.3%, n = 1 each) proposed a number of additional improvements, such as improved staff training, implementation of the protocol in all hospitals, preventative management with bed net insecticide, and no improvements required.

**Table 7:** Suggested improvements for the 2023 Sudan Malaria Treatment Protocol, priority training needs, and factors facilitating protocol implementation

		Responses	
		N	Percent
Suggested Improvements to the 2023 Protocol			
Suggested Improvements to The Protocol	Better supply chain management for medications	189	17.7
	Clearer treatment protocols for complicated cases	194	18.1
	Enhanced patient education materials	232	21.7
	Better staff training	1	0.1
	Don’t know	1	0.1
	Improved diagnostic testing procedures	218	20.4
	Updated medication guidelines	232	21.7
	Applying the protocol to all hospitals	1	0.1
	None	1	0.1
	Prophylactic management with bed net insecticide	1	0.1
Areas Requiring Additional Training	Data recording and reporting	215	21.4
	Logistics and supply chain management	164	16.3
	Don’t know	1	0.1
	Medication administration and dosage	196	19.5
	Patient management of severe malaria	192	19.1
	Microscopy and diagnostic techniques	238	23.7
Factors That Improve the Implementation of the 2023 Protocol:			
Suggested Improvements of Implementation	Better communication between healthcare providers	207	18.6
	More effective patient education	242	21.8
	Streamlined data reporting systems	160	14.4
	Don’t know	1	0.1
	Improved access to necessary medications and supplies	232	20.9
	Governorate insecticide program	1	0.1
	Increased staff training	268	24.1

Because participants could choose more than one recommended improvement, the overall percentage (288.4%) significantly surpasses 100%, suggesting that the majority of medical professionals identified several areas where the 2023 protocol could be improved.

Healthcare professionals' opinions of areas that need more resources or training for better implementation of the 2023 Sudan Malaria Treatment Protocol are shown in Table 7. 64.2% of respondents (n = 238) said that the areas that needed the most training were microscopy and diagnostic techniques. 58.0% (n = 215) identified data recording and reporting, 52.8% (n = 196) identified medication administration and dosage, and 51.8% (n = 192) identified patient management of severe malaria. Of the participants, 44.2% (n = 164) recognized supply chain management and logistics. The only participant who said they were unsure of which areas needed more training was one (0.3%).

Because participants could choose more than one area that needed training, the overall percentage (271.2%) significantly surpasses 100%, suggesting that the majority of healthcare professionals understood the need for improved training in a variety of areas.

The factors that healthcare professionals believe could enhance the 2023 protocol's implementation are shown in Table 7. 72.2% of respondents (n = 268) ranked increased staff training as the most important factor, followed by more effective patient education (65.2%, n = 242) and easier access to essential supplies and medications (62.5%, n = 232). 55.8% (n = 207) of participants reported improved provider-to-provider communication, and 43.1% (n = 160) reported more efficient data reporting systems. Only a small percentage of participants identified additional factors; one participant (0.3%) identified the governorate insecticide program and expressed uncertainty about which factors could enhance implementation.

## Discussion

Regarding formal training on the 2023 Sudan Malaria Treatment protocol, despite 67.9% of the participants reporting awareness of the 2023 protocol, only 24.3% received training while 69.5% have not received formal training and adherence and its findings are consistent with a study in northern Sudan in 2023 [5]. This demonstrates the gap between the awareness and the actual implementation of the protocol. This difference suggests that although information concerning the protocol has been shared, deeper training, understanding, and implementation remains limited. In addition, it emphasizes the importance of structured training to stave off inappropriate.

Due to limited training resources and the war that began in 2023 in Sudan, junior staff (82.7% of the participants lower than a 5-year-experience) might not be prioritized for training. Moreover, 21.6% of the participants were from rural areas.where there are limited training opportunities and these proportions are consistent with a study in Guinea [5]. Importantly, low formal training and the absence of a solid system to guide management and adherence to the protocol might have been a reason for the professionals to seek alternative plans; with 49.9% recommending rapid diagnostic tests, 39.4% relying on symptoms to clinically diagnose malaria as noted in the 2023 protocol [6] and only 0.3% recommending blood film as a diagnostic method for malaria less than what was mentioned in the Sudan protocol [6]. Hence, having a solid training program prioritizing the juniors will increase adherence to workflow and eliminate the gap.

On the other hand, regarding adherence to the 2023 Sudan Malaria protocol, even though about two thirds of the participants revealed their confidence in the effectiveness of the 2023 protocol, and emphasized their willingness to adopt it. Only less than half of them reported they always follow the protocol; while less than one third said they often do so. This explores the presence of barriers that hinder full adherence. This could be attributed to the knowledge gap in the professionals due to training insufficiency; as discussed previously. Moreover, challenges related to Sudan's healthcare system might be a reason as 53.37% reported a lack of resources (e.g., the absence of recommended IV drugs for severe malaria would impose professionals working around the protocol).

According to the results of the study, 93.5% of participants were able to identify Artemether-Lumefantrine as the first-line treatment and in line with the 2023 protocol while artemether-lumefantrine was the most commonly used treatment, with a lower percentage (66.7%) according to the Guinea study [4]. Despite this high level of agreement, there remains a significant gap, with 43.4% of practitioners still using IV Quinine for the treatment of severe malaria whilst the 2023 guidelines recommend the use of IV Artesunate as the preferred treatment.

Of the participants, 41.5% said they always adhere to the protocol when treating patients alone but only 22.9% of them reported so when collaborating with other healthcare workers. On the other hand, despite 48.79% of the patients were non-compliant to adopt the protocol, 64.96% of them also lost follow-up while adhering to the protocol. This reveals significant communication and workflow barriers between the co-workers in addition to, insufficient doctor to patient education and counseling regarding the diagnosis and importance of treatment. Sudan malaria protocol was last updated in 2023 in response to optimize therapeutic efficiency, therefore we believe problems related to Sudan's healthcare system should be addressed first as discussed followed by the formulation of solid training programs that could eliminate barriers from both system and individual levels which will consequently improve adherence to the protocol.

Even though the majority of health professionals demonstrated understanding of the protocol; yet, there remain unrecognised deficiencies in the training delivery that contribute to inadequacies in the protocol's implementation. Based on that, we recommend for the implementation of official training programs, not alone for Malaria Protocol 2023 but also for any newly implemented health guidelines in Sudan, emphasising the participation of the junior personnel and those in rural areas. The training programs should primarily focus the precision of diagnostic methods over dependence on clinical diagnosis, treatment guidelines, and also underscoring the significance of interprofessional collaboration, which will ultimately enhance protocol adherence and standardise malaria management nationwide.

We also advocate prioritising community-targeted awareness initiatives for both professionals and local communities, such as enhanced distribution of the protocol via the social media channels of federal and state health ministries to improve access to health information, the utilisation of scientifically explicit, patient-focused, and culturally acceptable educational posters and flyers in hospitals, primary health care institutions, and public facilities to enhance patient compliance.

Moreover, we advise health care providers to serve as conduits by disseminating information and teaching visitors at health facilities regarding the protocol, as well as providing counselling. Community-oriented awareness campaigns, outreach initiatives, and health promotion activities conducted by the FMOH in conjunction with local health organizations could also effectively address the deficiencies in knowledge, adherence, and follow-up within local communities.

Finally, while several impediments to protocol implementation were discovered, we advocate doing additional qualitative assessments and research to thoroughly investigate the obstacles impeding adherence to the guidelines, particularly in interprofessional contexts.

## Conclusion

Sudanese medical professionals demonstrated moderate-to-high awareness of the 2023 Sudan Malaria Treatment Protocol. However, formal training and consistent adherence remain suboptimal. Resource limitations and lack of structured training were the most frequently reported barriers. Strengthening dissemination strategies, training programs, and implementation support may improve adherence to protocol recommendations.

## Data Availability

Data will be available upon request.
